# Investigating Glycol-Split-Heparin-Derived Inhibitors of Heparanase: A Study of Synthetic Trisaccharides

**DOI:** 10.3390/molecules21111602

**Published:** 2016-11-23

**Authors:** Minghong Ni, Stefano Elli, Annamaria Naggi, Marco Guerrini, Giangiacomo Torri, Maurice Petitou

**Affiliations:** Istituto di Ricerche Chimiche e Biochimiche G. Ronzoni, srl, via G. Colombo 81, 20133 Milano, Italy; niminghong@ronzoni.it (M.N.); elli@ronzoni.it (S.E.); naggi@ronzoni.it (A.N.); guerrini@ronzoni.it (M.G.); torri@ronzoni.it (G.T.)

**Keywords:** heparanase, heparin, heparan sulfate, periodate oxidation, oligosaccharide synthesis, oligosaccharide conformation

## Abstract

Heparanase is the only known endoglycosidase able to cleave heparan sulfate. Roneparstat and necuparanib, heparanase inhibitors obtained from heparin and currently being tested in man as a potential drugs against cancer, contain in their structure glycol-split uronic acid moieties probably responsible for their strong inhibitory activity. We describe here the total chemical synthesis of the trisaccharide GlcNS6S-GlcA-1,6anGlcNS (**1**) and its glycol-split (gs) counterpart GlcNS6S-gsGlcA-1,6anGlcNS (**2**) from glucose. As expected, in a heparanase inhibition assay, compound **2** is one order of magnitude more potent than **1**. Using molecular modeling techniques we have created a 3D model of **1** and **2** that has been validated by NOESY NMR experiments. The pure synthetic oligosaccharides have allowed the first in depth study of the conformation of a glycol-split glucuronic acid. Introducing a glycol-split unit in the structure of **1** increases the conformational flexibility and shortens the distance between the two glucosamine motives, thus promoting interaction with heparanase. However, comparing the relative activities of **2** and roneparstat, we can conclude that the glycol-split motive is not the only determinant of the strong inhibitory effect of roneparstat.

## 1. Introduction

Heparanase is an endo-β-d-glucuronidase that cleaves the glycosaminoglycan chains of heparan sulfate at specific sites, thus modulating the biological function of this proteoglycan expressed at the cell surface of nearly all animal species [1]. Heparan sulfate and heparanase are involved in several pathological conditions, particularly tumor development [2], but also inflammation, diabetes, and atherosclerosis [3]. Heparan sulfate glycosaminoglycan is a linear polysaccharide containing alternating units of uronic acids (d-glucuronic or l-iduronic) and glucosamine, both bearing various substituent groups at various positions [4]. Heparanase degrades heparan sulfate through hydrolysis of the glycosidic linkage between selected glucuronic acid units and glucosamine [5,6,7]. The 3D structure of human heparanase has been recently reported [8].

**Figure 1:**
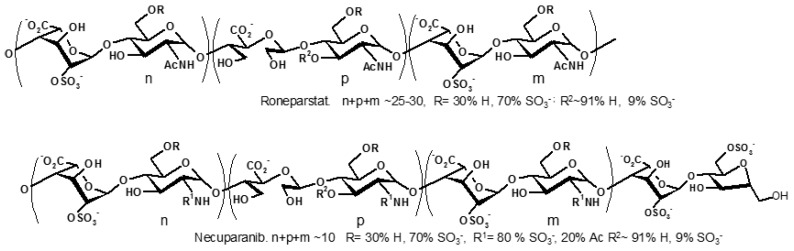
Schematic representation of roneparstat and necuparanib. The actual structure may retain the microheterogeneity of the original heparin and Low Molecular Weight Heparin.

Drugs targeting heparanase are actively looked for [9,10]. Among the known heparanase inhibitors roneparstat (SST0001, Sigma Tau Research) [11] and necuparanib (M 402, Momenta) [12], are the most advanced in clinical development (SST0001 currently in phase I in advanced multiple myeloma and M 402 in phase II for pancreatic cancer). Roneparstat is obtained from standard porcine mucosal heparin after total *N*-desulfation, *N*-acetylation, controlled periodate oxidation, and finally borohydride reduction (the sequence of the last two steps is referred to as glycol-splitting, abbreviated gs), whereas necuparanib is a low molecular weight heparin, resulting from depolymerization of heparin and further periodate oxidation and borohydride reduction. Schematic representations of roneparstat and necuparanib, both containing glycol-split uronic acid moieties, are depicted in Figure 1.

Several studies have been carried out to understand the structure-activity relationship of heparin for heparanase inhibition, including variation in the degree of 2-*O*-sulfation, 6-*O*-sulfation, *N*-sulfation, *N*-acetylation, glycol-split uronic acid [13,14]. These studies led to conclude that *O*-2-sulfation of IdoA and *O*-6 sulfation of GlcN were not essential for effective inhibition, and that one *N*-sulfate group per disaccharide units was in interaction with the enzyme [7]. It was also found that glycol-split of non-sulfated uronic acid dramatically increased the inhibitory activity [15]. Recent studies on the kinetics of heparanase inhibition by roneparstat suggest that it interacts with heparanase by multiple protein-ligand mode depending on the concentration of the inhibitor [16]. However the mechanism of action of both roneparstat and necuparanib at a molecular level is not clearly understood, particularly the role of the “glycol-split” uronic acid units that are key element in the structure must be clarified. Whether these units critically interfere with the active site of heparanase or merely serve for introducing conformational flexibility in the polysaccharide chains is still an open question. To document this aspect we have embarked on the chemical synthesis of homogenous heparan sulfate fragments and their “glycol-split” counterparts. Their activity as heparanase inhibitors will be tested and they will also be used for interaction studies, particularly by NMR. We report here on the synthesis, activity and conformation of a trisaccharide unit **1** and its glycol-split counterpart **2**.

## 2. Results and Discussion

### 2.1. Chemistry

**Scheme 1:**
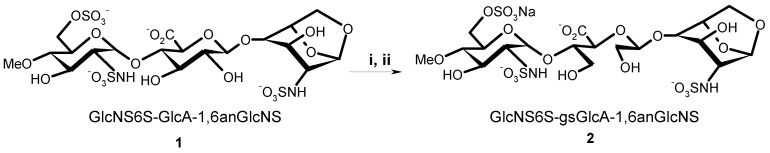
Conversion of **1** into **2**. Reagents and conditions: (i) NaIO_4_, H_2_O; (ii) NaBH_4_, 70%.

It should be underlined first that whereas all glucosamine units in roneparstat are *N*-acetylated, we decided to synthesize oligosaccharides containing *N*-sulfated-glucosamine, reflecting the situation in M402, with whom we expect a stronger interaction with heparanase. Indeed a stronger interaction with proteins is expected with *N*-sulfated glucosamine-containing oligosaccharides compared to their *N*-acetylated counterparts [7] (*N*-acetyl groups were introduced in roneparstat with a view to restrict undesired interactions with growth factors which is not an issue with short oligosaccharides, particularly if they do not contain 3-*O*-sulfated glucosamine units). From a synthesis standpoint, the amino group substituent is introduced during the last step of the synthesis, which makes the preparation of both *N*-acetylated and *N*-sulfated compounds very similar although more care must be taken for *N*-sulfated derivatives due to their higher sensitivity to acidic media. Thus, our primary target molecules were trisaccharide **1** (GlcNS6S-GlcA-1,6anGlcNS) and its derivative **2** (GlcNS6S-gsGlcA-1,6anGlcNS) that contains one single glycol-split glucuronic acid (gsGlcA) unit (Scheme 1). Note that a methyl substituent is present at position 4 of the non-reducing glucosamine in **1** to prevent cleavage of this unit by periodate during the conversion of **1** into **2**. The methyl groups also mimics the anomeric carbon of the next unit in a polysaccharide chain. In the framework of our whole project, fully protected 1,6-anhydro containing trisaccharides will be used as synthetic intermediates and, in passing, to settle the experimental conditions for periodate oxidation we decided to use a trisaccharide with a 1,6-anhydro-glucose ring at the reducing end (compound **1**).

**Scheme 2:**
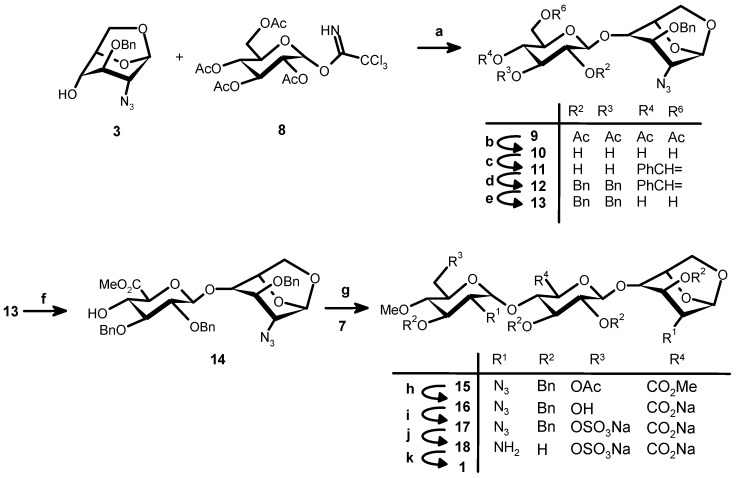
Synthesis of **1**. *Reagents and conditions*: (a) BF_3_:Et_2_O, 4 Å MS, CH_2_Cl_2_, −30 °C, 2 h, 77%; (b) 0.5 M MeONa, THF-MeOH, RT, 1 h; (c) PhCH(OMe)_2_, CSA, DMF, RT, 17 h, 56% (2 steps); (d) BnBr, NaH, DMF, RT, 2 h; (e) 70% aq. TFA, CH_2_Cl_2_, RT, 4 h; (f) (i) Dibromantin, TEMPO, CH_3_CN, RT, 2.5 h; (ii) CH_3_I, NaHCO_3_, DMF, RT, 16 h, 82% (4 steps); (g) TMSOTf, 4 Å MS, CH_2_Cl_2_, −20 °C, ~2 h, 58% (+ β-anomer 20%); (h) LiOH, H_2_O_2_, THF-MeOH, 16 h, 82%; (i) SO_3_:C_5_H_5_N, C_5_H_5_N, 55 °C, 2 h, 76%; (j) H_2_, 10% Pd/C, tBuOH-H_2_O, 60 h, 100%; (k) SO_3_:C_5_H_5_N, NaOH, RT, 16 h, 57%.

**Scheme 3:**
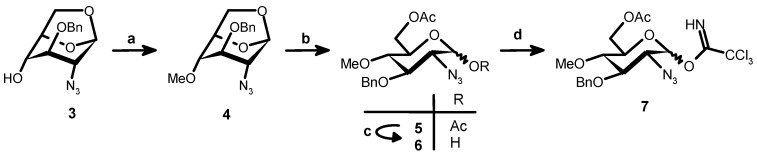
Synthesis of imidate **7**. *Reagents and conditions*: (a) CH_3_I, NaH, DMF, RT, 2 h, 100% (crude); (b) Ac_2_O-TFA, RT, 16 h, 100% (crude); (c) BnNH_2_, THF, RT, 3 h, 100% (crude); (d) Cl_3_CCN, CH_2_Cl_2_, Cs_2_CO_3_, RT, 3 h, 60% (4 steps).

Synthesis of the target trisaccharide **1** is depicted in Scheme 2 and Scheme 3. They were obtained by an adaptation of methods already used for this type of oligosaccharide [17]. A fully protected equivalent of **1** was prepared first with full orthogonal protection (**15**, Scheme 2) and then sequentially de-protected and substituted. We prepared first the known key derivative **3** [18] from commercially available levoglucosan (overall yield 21%), using the method of Oikawa et al. [19]. A first attempt to glycosylate **3** using tetraacetyl glucopyranose trichloroacetimidate [20] (**8**) in the presence of TMSOTf gave very little desired product **9**, together with a large quantity of a side product identified as 1,6-anhydro-2-azido-3-*O*-benzyl-4-*O*-acetyl-2-deoxy-β-d-glucopyranose. Its structure was confirmed by NMR and MS. Using BF_3_·Et_2_O as Lewis acid we could get a much improved yield of **9** (76%) that gave **11** after acetyl group removal and benzylidenation. Benzylation followed by cleavage of the benzylidene protection gave **13** [21] ready for selective oxidation of the primary alcohol by dibromantin-TEMPO. Methylation gave **14** (82% from **11**). 

The known imidate **7** [22] was obtained (Scheme 3) from **3** in 60% yield after methylation followed by acetolysis, selective anomeric deacetylation and treatment with trichloroacetonitrile in the presence of Cs_2_CO_3_. Coupling between **7** and **14** in dichloromethane in the presence of TMSOTf gave the trisaccharide **15** (58%) together with the β anomer (20%). Finally, starting from **15**, deacetylation, *O*-sulfation, hydrogenolysis and *N*-sulfation gave **1** (Scheme 2, 35.5%, 4 steps).

Periodate oxidation of **1** followed by borohydride reduction gave **2** (Scheme 1, 70.4%, 2 steps). The oxidation reaction was monitored by NMR spectroscopy (Supplementary Materials Figure S1), observing the disappearance of H-2 of GlcA at 3.44 ppm. We tested up to 20 equivalents of sodium periodate with respect to the diol. Finally, using four equivalents a 96% conversion from diol to dialdehyde was obtained within 4 hours and the formation of side products was avoided.

### 2.2. Heparanase Inhibition Assay In Vitro

Heparanase inhibitory activity of compounds **1** and **2** in comparison with roneparstat was determined in vitro according to Hammond et al. [23] using fondaparinux as a substrate. Upon heparanase cleavage fondaparinux yields a trisaccharide and a disaccharide that can be quantitatively assessed using the tetrazolium salt WST-1.

**Figure 2:**
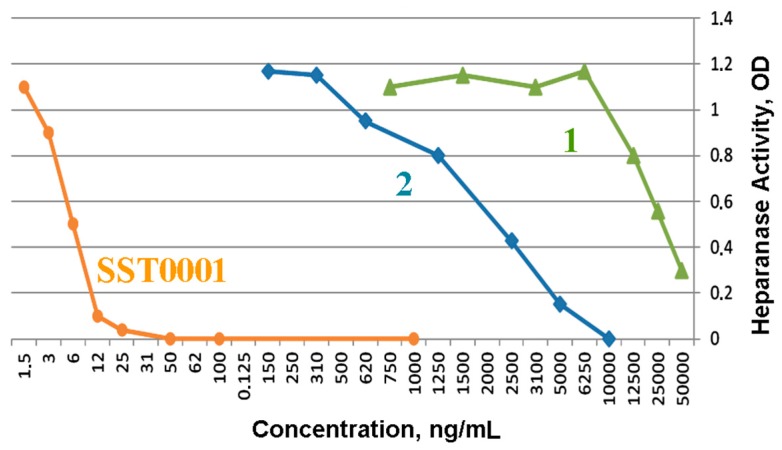
Dose-dependent inhibition of heparanase activity by SST001 (orange circles, IC_50_: 6 ng/mL); compound **2** (blue diamonds, IC_50_: 2 µg/mL) and compound **1** (green triangles, IC_50_: 30 µg/mL).

The results are shown in Figure 2. As expected, compound **2** (IC_50_ 2 µg/mL) was found to be one order of magnitude more potent than its analog **1** (IC_50_ 30 µg/mL), with both compounds being much less potent than roneparstat (IC_50_ 6 ng/mL).

### 2.3. Conformation Characterization

To understand the role of glycol-split units in the interaction with heparanase we performed molecular modeling of **1** and **2** and compared the models to experimental NMR data. The models were obtained using a molecular dynamic approach including a variable temperature “*enhanced sampling*” procedure, no constraint was applied during the simulation.

Considering the monosaccharide residues involved in **1** and **2**, while the conformation of pyranose rings in glycosaminoglycans has been largely studied [24,25], the conformation of glycol-split units particularly glycol-split glucuronic acid has been until now poorly documented. In one report [26] the authors applied a semi-quantitative interpretation of NOESY data obtained on glycol-split partially *O*-desulfated pig mucosal heparin (the uronic acid content of which was: glucuronic 20%, unsulfated iduronic 30%, sulfated iduronic 50%). A second report dealt with the conformation glycol-split iduronic acid (gsIdoA) in a heparin-like hexasaccharide bound to Fibroblast Growth Factor 2 (FGF-2). Greater flexibility of the molecule was observed, in comparison to a hexasaccharide containing intact iduronic acid [27], suggesting that glycol splitting induces a drastic divergence of the GAG chain from the propagation required for setting in the basic canyon generated by FGF/FGF-receptor assemblies. However an in depth study of the conformation of glycol-split glucuronic acid (gsGlcA) has not been reported.

**Figure 3:**
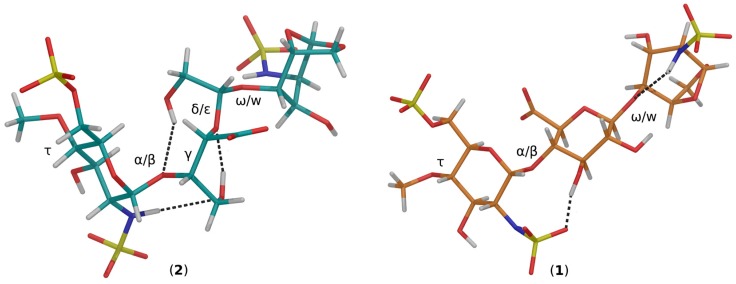
Trisaccharide predicted conformation for compound **2** and compound **1** adjusting the backbone dihedral angles: τ, α/β, γ, δ/ε, ω/w for the former and τ, α/β, ω/w for the latter, in accord to the average values estimated using the MD simulation sampling procedure (material and methods). Possible extra-residue (GlcA) hydrogen bonds, contributing to **2** and **1** conformation stability, are underlined by dashed lines joining donor and acceptors atoms.

We thus paid special attention to the backbone conformation of **2** that can be described by eight consecutive dihedral angles, from the non-reducing to the reducing end: τ, α/β, γ, δ/ε, ω/w. For **1** only five torsional angles are involved: τ, α/β, ω/w (Figure 3). For both **1** and **2**, α/β and ω/w characterize the classical glycosidic linkages, while τ describes the methoxy group conformation. The angles γ, and δ/ε, characterize additional degree of freedom that appear upon cleavage of the glucuronate ring.

Conformational sampling was done with temperature progressively increasing from 300 to 400 K, and decreasing from 400 to 300 K (Supplementary Materials Table S1); the last MD simulation step at 300 K allowed average dihedral angle calculation. Two initial conformations for each glycan were used which lead to the same final conformation showing the reliability of the method (Supplementary Materials Table S2). The dihedrals angle pairs: α/β, δ/ε, ω/w are reported in Ramachandran plots (Supplementary Materials Figure S2), while γ is reported as a function of time as it resembles an aliphatic chain torsional degree of freedom (H-C(R,R′)-C(R′′,R′′′)-H). The yellow stars in Ramachandran plots (Supplementary Materials Figure S2) indicate the estimated most probable states.

As shown in Figure 3, at the end of the modeling process the pyranose rings in **1** and **2** maintain the expected classical conformations, namely ^4^*C*_1_ for GlcNS6S and GlcA and ^1^*C*_4_ for 1,6anGlcNS, which is also in agreement with the ^1^H-NMR ^3^*J* inter-proton coupling constants observed (see the Materials and Methods section).

Regarding the interglycosidic angles, similar α/β distributions were found for **1** and **2** (Supplementary Materials Figure S2) but they have different ω/w distribution, **2** showing two accessible states (**A** and **B**) as opposed to a single state in **1**. As discussed below, only conformation **A** fits the experimental NOE data. The estimated backbone torsional angles of **1** and **2** were used to refine the models initially built. The refined conformations are displayed (Figure 3) where only the A conformer of **2** is shown. In contrast to GlcA in **1**, where OH-2′ and OH-3′ are in trans diequatorial orientation, in compound **2** cleavage of the C-2/C-3 bond allows OH-2′ and OH-3′ to be oriented at the opposite side of the molecule backbone (Figure 3). Interestingly, in **2** the most populated γ angle (approx. −60°) allows the highest distances between the carboxylate of gsGlcA and the two sulfate groups of the next GlcNS6S residue, while the greater flexibility of gsGlcA, allows a smaller trisaccharide “end-to-end” distance (between C-4 of GlcNS6S and C-1 of 1,6anGlcNS) 10.4 Å in **2**, vs. 12.4 Å in **1** (Figure 3).

The model (Maestro graphical interface, see material and methods) allows prediction of intra-molecular hydrogen bonds (Figure 3). In **1**, two bonds are predicted, the first one between an oxygen atom of the *N*-sulfate of GlcNS6S (acceptor) and OH-3 of GlcA (donor), and the second one between the GlcA-1,6anGlcNS interglycosidic oxygen (acceptor) and the NH of 1,6anGlcNS (donor). In **2**, three bonds are predicted: first between NH of GlcNS6S (donor) and OH-3 of gsGlcA (acceptor), second between OH-3 of gsGlcA (donor) and O-5 of gsGlcA (acceptor) and third between OH-2 of gsGlcA (donor) and the interglycosidic GlcNS6S-gsGlcA oxygen (acceptor).

The models obtained were then confronted to experimental NMR data. Regarding chemical shifts, signals of the HSQC spectra of **1** and **2** (Supplementary Materials Figure S3) support their modelled conformation. Thus, the anomeric proton of GlcNS6S in **1** is more de-shielded than in **2** (5.61 vs. 5.34 ppm) which can be explained by the shorter distance between this proton and the carboxyl group of the adjacent uronic acid, smaller in **1** (4.6 Å) than in **2** (5.1 Å). NMR HSQC experiment data also support the above H-bond network in **2**, showing H-2a and H-2b resonances as superimposed signals centered at 3.66 ppm, in accord with a partially impaired rotation of the CH_2_OH group around the C-1/C-2 bond. In contrast, the H-3a and H-3b (3.92 and 3.80 ppm) show diastereotopic effect, being separated by more than 0.1 ppm, suggesting a higher impairment in the rotation of CH_2_OH around C-3/C-4 in gsGlcA.

The strength of various hydrogen bonds can be compared using ^1^H-NMR at variable temperature. The chemical shift of the exchangeable protons in **2**: NH of the two glucosamines, OH-2′ and OH-3′ of gsGlcA, were measured at temperature from 5 to 35 °C. The chemical shifts (ppm) were fitted to the temperature (K) using a linear regression model and the temperature coefficient was calculated (see experimental part). The temperature coefficient reflects the extent to which NH and OH protons are protected from exchange through hydrogen bonding. Temperature coefficients showed different behavior between NHSO_3_ and OH protons, the former had a value of −5.7/−8.1 ppb/K while the second had a value of −9.8/13.0 ppb/K (Supplementary Materials Figure S4 and Table S3). We can conclude from the present experiment that the hydrogen bonds involving NH of the glucosamines are stronger than those involving the OH′ groups. Additionally, the bond involving OH-3′ appears slightly stronger than that involving OH-2′, in agreement with the intra-molecular hydrogen bond network predicted, and the interpretation of the HSQC spectra.

**Figure 4:**
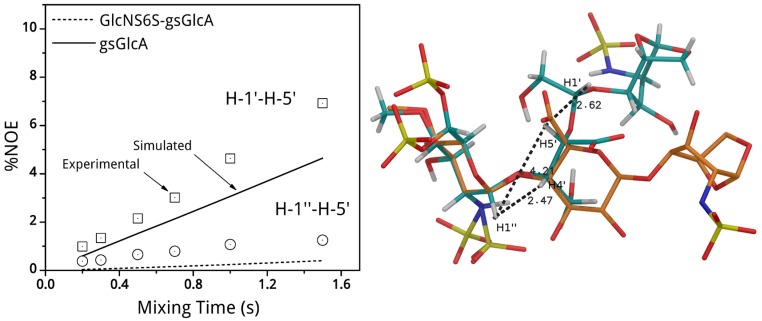
(**left**) Selected experimental and simulated NOEs signals build up curves for compound **2** measured between protons H-1′′/H-5′ across the glycosidic linkage GlcNS6S-gsGlcA (empty circles, dotted line), and between H-1′/H-5′ inside the gsGlcA residue (empty squares, continuous line). Symbols and lines indicate experimental and simulated NOEs respectively; (**right**) Compound **2** and compound **1** predicted conformations, represented by cyan and orange tubes respectively are superposed by the GlcNS6S residue. Selected distances: H-1′′/H-4′, H-1′′/H-5′, and H-1′/H-5′ are expressed in Ǻ.

The models were also used to simulate selected intra and inter-residue 2D NOE signals to be compared with the corresponding experimental values. These latter were determined as build-up functions of the mixing time between 0.2 s to 1.0 s for the trisaccharide **1** or 0.2 s to 1.5 s, for the glycol-split **2**. Comparison of experimental and calculated NOEs for the two possible models obtained for **2** (Supplementary Materials Figure S2A,B) shows that conformation **A** gives NOEs in better agreement than **B** for the H-1′/H-4 pair and particularly for the H-1′/H-5 pairs (Supplementary Materials Figure S6). Only this conformation was taken into account for comparison of other experimental and calculated NOEs. Experimental intra-residue NOEs for H-1′′/H-2′′ and H-1/H-2 in **2**, measured at different mixing time, were used to tune the correlation time using NOEPROM software. The A conformation of **2** (Supplementary Materials Figure S2) was used to calculate NOEs of various pairs of protons and the results were compared with the experimental data. A good agreement was observed for all protons pairs that could be experimentally tested: H-1′′/H-5′, H-1′′/H-4′, H-1′/H-4, H-1′/H-5 (Figure 4, see also Supplementary Materials Figure S6).

The optimized conformation of the trisaccharide **1** is supported by the good agreement between the measured and the calculated H-1′′/H-4′ and H-1′-H-4 inter-residue NOEs, validating the values obtained for the interglycosidic dihedral angles (Supplementary Materials Figure S6). Due to several signal overlap and strong couplings, no other NOE build-up curves could be undoubtedly measured for **1**.

Finally, the model predicts that **1** and **2** having the same total charge, differ in charge distribution, highlighted by dissimilar electric dipole moment (Supplementary Materials Figure S7). A possible explanation could be the higher flexibility of the gsGlcA residue allowing a better neutralization of the atomically distributed charges, resulting in a smaller dipole moment vector length in **2** which could be the basis for a significantly different intermolecular force behaviour. 

## 3. Materials and Methods

### 3.1. General Information

All reagents were purchased from Sigma-Aldrich (Milan, Italy) and Iris Biotech GmbH (Marktredwitz, Germany). Solvents were freshly distilled before to use. All reactions were carried out under a nitrogen atmosphere if necessary. Flash chromatography was performed on silica gel (230–400 mesh, Merck, Darmstadt, Germany). TLC was carried out on silica gel plate (Merck 60, F_254_) and detected visually by ultraviolet irradiation (254 nm) or by detected with spray (10% conc. sulphuric acid, heating at 130 °C. NMR spectra were recorded on a Bruker Avance 500 MHz or Avance 600 MHz spectrometer (Bruker, Karlsruhe, Germany). All values were reported in ppm (δ) downfield from solvent. HRMS was obtained with MicrOTOF-Q (Bruker Daltonics, Brema, Germany). LC-MS was performed on an Ultimate 3000 HPLC-UV system (Dionex, Rodano, Italy) coupled to an Esi-Q-TOF-MS MicrOTOF-Q (Bruker Daltonics). Ion-pair reversed-phase separation was carried out on a Kinetex C18 column (2.1 mm × 100 mm, 2.6 μm, 100 Å, Phenomenex, Bologna, Italy). A binary solvent system plus 10 mM DBU and AcOH was used for gradient elution.

### 3.2. Syntheses

*1,6-Anhydro-2-azido-3-O-benzyl-2-deoxy-β-d-glucopyranose* (**3**). Compound **3** was synthesized in 7 steps (21% overall yield) from commercially available 1,6-anhydro-β-d-glucopyranose following the method of Oikawa et al. [19].

*1,6-Anhydro-2-azido-3-O-benzyl-2-deoxy-4-O-methyl-β-d-glucopyranose* (**4**). Methyl iodide (0.5 mL, 8.6 mmol) was added to a cooled (0 °C) solution of **3** (2 g, 7.2 mmol) in dry DMF (32 mL). Sodium hydride 50% in oil (0.5 g, 10.8 mmol) was then introduced in portions and the reaction mixture was stirred at RT for 2 h. After cooling to 0 °C NaH in excess was destroyed by methanol (20 mL). After evaporation under vacuum EtOAc (100 mL) and water (50 mL) were added. The aqueous phase was washed with EtOAc (50 mL) and the combined organic phases were washed with brine (50 mL) and dried (Na_2_SO_4_). After evaporation crude **4** (2.3 g) was obtained as syrup and used for the next step without further purification. ESIMS *m*/*z*: [M + Na]^+^: 314.0373, [M + K]^+^: 330.0077. Calculated for C_14_H_17_N_3_O_4_ + Na: 314.1117, C_14_H_17_N_3_O_4_K: 330.0856.

*6-O-Acetyl-2-azido-3-O-benzyl-2-deoxy-4-O-methyl-β-d-glucopyranose* (**6**). A mixture of trifluoroacetic acid (5.5 mL, 72 mmol) in acetic anhydride (68 mL, 721 mmol) was added to **4** (2.3 g, 7.2 mmol) and the resulting mixture was stirred overnight at room temperature. After evaporation under vacuum and co-evaporation with toluene the residue was dissolved in CH_2_Cl_2_ (100 mL), washed with saturated aqueous NaHCO_3_ (40 mL) and brine (50 mL × 1). After drying (Na_2_SO_4_) and evaporation **5** was obtained (2.93 g, 96.6%) and used for the next step without further purification. ESIMS *m*/*z*: [M + Na]^+^: 416.0497, [M + K]^+^: 432.0180. Calculated for C_18_H_23_N_3_O_7_Na: 416.1434, C_18_H_23_N_3_O_7_K: 432.1173.

Benzylamine (1.4 mL, 12.8 mmol) was added to a solution of **5** (2.7 g, 6.4 mmol) in dry THF after stirring for 3 h at RT the solvent was evaporated under vacuum. Ethyl acetate (100 mL) was added to the residue and the solution was washed with 1 M HCl (20 mL). The aqueous phase was washed with ethyl acetate (50 mL) and the combined organic phases were washed with H_2_O (20 mL), brine (20 mL) and dried (Na_2_SO_4_). After evaporation **6** (3.24 g) was obtained as a brown syrup and used as such in the next step. ESIMS *m*/*z*: [M + Na]^+^: 374.0518, [M + K]^+^: 390.0207. Calculated for C_16_H_21_N_3_O_6_Na: 374.1328, C_16_H_21_N_3_O_6_K: 390.1062. 

*6-O-Acetyl-2-azido-3-O-benzyl-2-deoxy-4-O-methyl-1-O-trichloroacetimidoyl-d-glucopyranose* (**7**). Cs_2_CO_3_ (1.86 g, 5.7 mmol) was added at 0 °C to a solution under nitrogen atmosphere of crude **6** (3.24 g, 6.4 mmol) and Cl_3_CCN (3.83 mL, 38.2 mmol) in CH_2_Cl_2_ (63 mL, dried over molecular sieves). After stirring at RT for 3 h the solution was filtered through Celite and the filter pad was washed with CH_2_Cl_2_ (60 mL). The dichloromethane solution was washed with H_2_O, brine and dried (Na_2_SO_4_). After concentration and flash chromatography (98:2–95:5 CH_2_Cl_2_–EtOAc) **7** (α:β ratio 1:6) was obtained (1.88 g; 59.9%). ESIMS *m*/*z*: [M + Na]^+^: 516.9294. Calculated for C_18_H_21_Cl_3_N_4_O_6_Na: 517.0418.

*(2,3,4,6-tetra-O-acetyl-β-d-glucopyranosyl)-O-(1→4)-1,6-anhydro-2-azido-3-O-benzyl-2-deoxy-β-d-gluco-pyranose* (**9**). A cold (−30 °C) solution of **3** (5 g, 18.21 mmol) and **8** (13.45 g, 27.31 mmol, 1.5 eq) in dry CH_2_Cl_2_ (180 mL) containing 4 Å molecular sieves (5 g, previously activated at 400 °C for 4 h), was stirred at RT for 15 min under nitrogen atmosphere. After cooling at −30 °C, a solution of BF_3_:Et_2_O (0.75 mL, 5.89 mmol, 0.3 eq) in CH_2_Cl_2_ (225 mL) was added dropwise over 30 min. The temperature was slowly raised to room temperature (in about 45 min). After 2 h the reaction mixture was neutralized by TEA. After evaporation under vacuum and flash chromatography (85:15 hexane–EtOAc) **9** was obtained (8.49 g, 76.7%) as a glass. ^1^H-NMR (500 MHz, CDCl_3_) δ: 7.36–7.31 (m, 5H, Ph), 5.45 (s, 1H, H-1), 5.21 (t, 1H, *J* = 9.5 Hz, H-3′), 5.11 (t, 1H, *J* = 9.5 Hz, H-4′), 5.01 (dd, 1H, *J* = 9.5 and 8.0 Hz, H-2′), 4.74 (d, 1H, *J* = 8.0 Hz, H-1′), 4.65 (s, 2H, *CH*_2_Ph), 4.58 (d, 1H, *J* = 5.0 Hz, H-5), 4.20 (dd, 1H, *J* = 12.5 and 4.5 Hz, H-6′a), 4.14 (m, 1H, H-6′b), 4.09 (d, *J* = 7.5 Hz, 1H, H-6a), 3.79 (s, 1H, H-3), 3.77 (br s, 2H, H-4 + H-6b), 3.67–3.63 (m, 1H, H-5′), 3.23 (s, 1H, H-2), 2.04 (s, 6H, *CH*_3_CO), 2.03 (s, 3H, *CH*_3_CO), 2.01 (s, 3H, *CH_3_*CO). ^13^C-NMR (CDCl_3_) δ: 170.47, 170.27, 169.26, 169.16 (4 C=O), 137.33, 128.52, 128.03, 127.65 (Arom. Ph) 100.69 (C-1), 99.36 (C-1′), 77.22 (C-4), 75.96 (C-3), 73.72 (C-5), 72.73 (C-3′), 72.45 (C-5′), 72.14 (*CH*_2_Ph), 71.27 (C-2′), 68.21 (C-4′), 65.05 (C-6′), 61.81 (C-6), 59.47 (C-2), 20.54 (*CH_3_*CO). ESIMS *m*/*z*: [M + Na]^+^: 630.0705, [M + K]^+^: 646.0700. Calculated for C_27_H_33_N_3_O_13_Na: 630.1905, C_27_H_33_N_3_O_13_K: 646.1644.

*β-d-Glucopyranosyl-O-(1→4)-1,6-anhydro-2-azido-3-O-benzyl-2-deoxy-β-d-glucopyranose* (**10**). A sodium methanolate solution (0.5 M in methanol, 45 mL, 22.5 mmol) was slowly added at 0 °C to a solution of **9** (9.12 g, 15.01 mmol) in THF–methanol (1:1, 98 mL). After 1 h at RT Amberlite^®^ IR-120 (H^+^-form, previously washed with H_2_O and MeOH) was added until pH 2. After removal and washing of the resin the solution was concentrated to give **10** as a white solid used as such in the next step. ESIMS *m*/*z*: [M + Na]^+^: 462.5705, [M + K]^+^: 478.5600. Calculated for C_19_H_25_N_3_O_9_Na: 462.1488, C_19_H_25_N_3_O_9_K: 478.1228.

*(4,6-O-Benzylidene-l-β-d-glucopyranosyl)-O-(1→4)-1,6-anhydro-2-azido-3-O-benzyl-2-deoxy-β-d-gluco-pyranose* (**11**). Camphorsulfonic acid (345 mg, 1.5 mmol) followed by benzaldehyde dimethylacetal (45.2 mL, 300 mmol) were added at RT, under a nitrogen atmosphere, to a solution of crude **10** (6.6 g, 15 mmol) in dry DMF (56 mL). After overnight stirring, a saturated aqueous solution of NaHCO_3_ (30 mL) was added. After dilution with EtOAc (250 mL) the organic phase was washed with brine, dried (Na_2_SO_4_), and concentrated. The crude product was purified by flash chromatography (1:1 CH_2_Cl_2_–EtOAc) to give **11** as white solid (4.43 g, 56.3% from **9**). ^1^H-NMR (500 MHz, CDCl_3_) δ: 7.51–7.32 (m, 10H, aroma), 5.55 (s, 1H, acetal), 5.53 (s, 1H, H-1), 4.71 (d, *J* = 5.5 Hz, 1H, H-5), 4.67, 4.60 (AB system, *J* = 12 Hz, 2H, *CH*_2_Ph), 4.53 (d, *J* = 7.5 Hz, 1H, H-1′), 4.27–4.24 (m, 1H, H-6′), 4.17 (d, *J* = 7.0 Hz, 1H, H-6), 3.83 (d, *J* = 5.5 Hz, 1H, H-3), 3.82 (s, 1H, H-4), 3.81 (m, 1H, H-6), 3.81 (m, 1H, H-3′), 4.25–3.76(m, 1H, H-6′), 3.61–3.59 (m, 1H, H-2′), 3.59–3.56 (m, 1H, H-4′), 3.43–3.38 (m, 1H, H-5′), 3.22 (s, 1H, H-2). ^13^C-NMR (CDCl_3_) δ: 137.3–127.6 (Bn), 109.2 (*CH* Acetal), 102.2 (C-1′), 102.1 (C-1), 80.5 (C-4′), 77.3 (C-3), 75.6 (C-4), 74.3 (C-2′), 72.8 (*CH*_2_Ph), 68.7 (C-6′), 66.8 (C-5′), 65.2 (C-6), 59.2 (C-2). ESIMS *m*/*z*: [M + Na]^+^: 550.0657, [M + K]^+^: 566.0370 calculated for C_26_H_29_N_3_O_9_Na: 550.1796, C_26_H_29_N_3_O_9_ K: 566.1535.

*(2,3-di-O-Benzyl-4,6-O-benzylidenel-β-d-glucopyranosyl)-O-(1→4)-1,6-anhydro-2-azido-3-O-benzyl-2-deoxy-β-d-glucopyranose* (**12**). Benzyl bromide (2.4 mL, 20.31 mmol) was added under nitrogen atmosphere to a solution of 11 (4.12 g, 7.81 mmol) in dry DMF (27 mL). After cooling (0 °C) NaH (1.50 g, 50% dispersion in oil, 31.24 mmol) was added in portions. After stirring at RT for 2 h the solution was cooled to 0 °C and NaH in excess was destroyed by slow addition of methanol (25 mL). After evaporation in vacuo EtOAc (150 mL) and H_2_O (50 mL) were added. The aqueous phase was extracted by EtOAc (50 mL) and the combined organic solutions were washed with brine (50 mL) dried (Na_2_SO_4_) and concentrated to dryness to give crude 12 (6.3 g) as a syrup used as such in the next step. ESIMS *m*/*z*: [M + Na]^+^: 730.1302, [M + K]^+^: 746.0999. Calculated for C_40_H_41_N_3_O_9_Na: 730.2735, C_40_H_41_N_3_O_9_K: 746.8676. 

*(2,3-di-O-Benzyl-β-d-glucopyranosyl)-O-(1→4)-1,6-anhydro-2-azido-3-O-benzyl-2-deoxy-β-d-gluco-pyranose* (**13**). An aqueous TFA solution (70%, 12 mL) was added to a solution of **12** (6.3 g, 7.81 mmol) in CH_2_Cl_2_ (270 mL). After stirring at RT for 4 h, aqueous NaHCO_3_ (saturated solution, 200 mL) was added. The aqueous phase was washed with EtOAc (50 mL × 1). Combined organic phases were washed once by brine, dried (Na_2_SO_4_), filtered and concentrated to give **13** (6.11 g) as colorless syrup.

ESIMS *m*/*z*: [M + Na]^+^: 642.1171, [M + K]^+^: 658.0868 calculated for C_33_H_37_N_3_O_9_Na: 642.2422, C_33_H_37_N_3_O_9_K: 658.2161.

*(Methyl 2,3-di-O-benzyl-β-d-glucopyranosyluronate)-O-(1→4)-1,6-anhydro-2-azido-3-O-benzyl-2-deoxy-β-d-glucopyranose* (**14**). TEMPO (122 mg, 0.781 mmol) and 1,3-dibromo-5,5-dimethyl hydantin (Dibromantin, 4.47 g, 15.62 mmol) were added to a solution of crude **13** (~7.81 mmol) in CH_3_CN (260 mL and 1% NaHCO_3_ aqueous solution (260 mL) and stirred at RT for 2.5 h. pH = 8. 1 M Na_2_S_2_O_3_ aqueous solution was added until the yellow colour was disappeared. At 0 °C 1 N H_2_SO_4_ solution was added to pH = 2. 150 mL of EtOAc was added. After two phases were separated, aqueous phase was extracted by EtOAc (120 mL × 2). Combined organic phases were dried over Na_2_SO_4_, filtered and concentrated to give crude uronic acid (7 g) directly for methylation. The above crude uronic acid (7 g,) was dissolved in dry DMF (76 mL). Solid NaHCO_3_ (6.56 g, 78.10 mmol, 10 eq) and iodomethane (4.86 mL, 78.10 mmol, 10 eq) were added under a nitrogen atmosphere and stirred at RT overnight. The reaction mixture was diluted with EtOAc (250 mL) and H_2_O (120 mL). After the two phases were separated, the aqueous phase was extracted with EtOAc (100 mL × 1). The combined organic phases were washed with 1 M Na_2_S_2_O_3_ (80 mL × 1) and brine (80 mL × 1). The crude product (6.16 g) was submitted to flash chromatography (hexane–EtOAc 6:4) to give compound **14** (4.13 g, yield: 81.8% of 4 steps) as a crystalline glass. ^1^H-NMR (500 MHz, CDCl_3_) δ: 7.39–7.28 (m, 15H, 3Ph), 5.52 (s, 1H, H-1), 5.01, 4.74 (AB system, *J* = 11.0 Hz, 2H, *CH*_2_Ph), 4.91, 4.83 (AB system, *J* = 11.5 Hz, 2H, *CH*_2_Ph), 4.67, 4.64 (AB system, *J* = 11.5 Hz, 2H, *CH*_2_Ph), 4.60 (d, *J* = 5.0 Hz, 1H, H-5), 4.57 (d, *J* = 7.5 Hz, 1H, H-1′), 4.05 (d, *J* = 7.0 Hz, 1H, H-6), 3.95 (t, *J* = 1.5 Hz, 1H, H-3), 3.90 (t, *J* = 9.5 Hz, 1H, H-4′), 3.79–3.75 (m, 1H, H-6), 3.78 (s, 1H, H-4), 3.78 (t, *J* = 10.0 Hz, 1H, H-5′), 3.76 (s, 3H, *CH_3_*O), 3.54–3.52 (m, 1H, H-2′), 3.51–3.49 (m, 1H, H-3′), 3.21 (s, 1H, H-2). ^13^C-NMR (CDCl_3_) δ: 169.39 (CO), 138.44,138.22,137.60 (Ph), 128.52, 128.49, 128.42, 128.38, 127.92, 127.62 (Ph), 103.91 (C-1′), 100.71 (C-1), 83.02 (C-3′), 80.78 (C-2′), 77.85 (C-3), 75.42 (C-4), 75.23, 74.42 (*CH*_2_Ph), 74.15 (C-5), 72.63 (C-5′), 71.56 (C-4′), 64.97 (C-6), 59.84 (C-2), 52.66 (*CH*_3_O). ESIMS *m*/*z*: [M + Na]^+^: 670.1001, [M + K]^+^: 686.068. Calculated for C_34_H_37_N_3_O_10_ + Na: 670.2371, C_34_H_37_N_3_O_10_K: 686.2111.

*(6-O-Acetyl-2-azido-3-O-benzyl-2-deoxy-4-O-methyl-α-d-glucopyranosyl)-O-(1→4)-(methyl 2,3-di-O benzyl-β-d-glucopyranosyluronate)-O-(1→4)-1,6-anhydro-2-azido-3-O-benzyl-2-deoxy-β-d-glucopyranose* (**15**). Compound **14** (1.679 g, 2.44 mmol), **7** (1.45 g, 2.928 mmol, 1.3 eq) and 4 Å molecular sieves (1.6 g) were stirred in dry DCM at RT for 1 h under nitrogen atmosphere. After cooling (−20 °C), TMSOTf (0.106 mL, 0.2 eq respect to donor) in DCM (16 mL) was added dropwise over 30 min. A precipitate formed and dissolved again. Additional compound **7** (0.19 g, 0.3 eq) and TMSOTf (0.025 mL) were added and after 15 min at −20 °C the mixture was allowed to warm up and maintained at RT for 1 h. TEA was added till pH 7. After filtration and evaporation purification was performed by flash chromatography (CH_2_Cl_2_–EtOAc, 95:5 to 92:8) to give **15** (1.387 g, 58%) and its beta anomer (0.465 g, 19.5%). ^1^H-NMR (500 MHz, CDCl_3_) (α anomer) δ: 7.4–7.26 (m, 20H, arom. Ph), 5.54 (d, *J* = 3.75 Hz, 1H, H-1′′), 5.50 (s, 1H, H-1), 5.04, 4.70 (AB system, *J* = 10.8 Hz, 2H, *CH*_2_Ph), 5.03, 4.82 (AB system, *J* = 10.8 Hz, 2H, *CH*_2_Ph), 4.85 (s, 2H, *CH*_2_Ph), 4.63, 4.60 (AB system, *J* = 10.8 Hz, 2H, *CH*_2_Ph), 4.61 (d, *J* = 7.7 Hz, 1H, H-1′), 4.59 (s, 1H, H-5), 4.26 (br s, 2H, H-6′′a + H-6′′b), 4.12 (t, *J* = 9.1 Hz, 1H, H-4′), 4.07 (d, *J* = 7.4 Hz, H-6a), 3.92 (d, *J* = 9.6 Hz, 1H, H-5′), 3.88 (br s, 1H, H-3′′), 3.79–3.74 (m, 4H, H-3 + H-4 + H-3′ + H-6b), 3.74 (s, 3H, Me ester), 3.63 (t, *J* = 7.7 Hz, 1H, H-2′), 3.50 (s, 3H, OMe), 3.48 (br s, 1H, H-5′′), 3.22–3.19 (m, 3H, H-2 + H-2′ + H-4′′), 2.10 (s, 3H, C*H*_3_CO). ^13^C-NMR (CDCl_3_) (α anomer) δ: 170.7 (C=O, acetyl), 168.3 (C=O, Me ester), 138.1–137.4 (Bn), 128.1–127.2 (Bn), 103.3 (C-1′), 100.7 (C-1), 97.5 (C-1′′), 83.8 (C-3′), 81.4 (C-2′), 80.0 (C-4′′), 79.5 (C-4), 77.5 (C-3′′), 76.7 (C-3), 75.1 (C-5), 74.3 (C-4′), 74.2 (C-5′), 75.3–74.2 (*CH*_2_, Bn), 72.7 (CH_2_, Bn), 69.7 (C-5′′), 65.0 (C-6), 63.0 (C-2′′), 62.3 (C-6′′), 60.8 (OMe), 59.9 (C-2), 52.7 (*CH*_3_, Me ester), 20.8 (*CH*_3_CO). ESIMS *m*/*z*: [M + Na]^+^: 1003.1659, [M + K]^+^: 1019.1354 calculated for C_50_H_56_N_6_O_15_Na: 1003.3696, C_50_H_56_N_6_O_15_K: 1019.3435. ^1^H-NMR (500 MHz, CDCl_3_) (β anomer) δ: 7.37–7.24 (m, 20H, 4Ph), 5.49 (s, 1H, H-1), 4.98, 4.72 (AB system, *J* = 11.4 Hz, 2H, *CH*_2_Ph), 4.91, 4.66 (AB system, *J* = 10.7 Hz, 2H, *CH*_2_Ph), 4.83, 4.78 (AB system, *J* = 11.3 Hz, 2H, *CH*_2_Ph), 4.64, 4.60 (AB system, *J* = 11.3 Hz, 2H, *CH*_2_Ph), 4.57 (d, *J* = 7.5 Hz, 1H, H-1′), 4.57 (d, *J* = 7.5 Hz, 1H, H-5), 4.42 (d, *J* = 7.6 Hz, H-1′′), 4.20 (m, 1H, H-4′), 4.16 8d, *J* = 12.5 Hz, 1H, H-6′′a), 4.12 (dd, *J* = 12.5 and 4.6 Hz, 1H, H-6′′b), 4.05 (d, *J* = 7.9 Hz, 1H, H-6a), 3.94 (d, *J* = 9.7 Hz, 1H, H-6a), 3.94 (d, *J* = 9.7 Hz, 1H, H-5′), 3.88 (br s, 1H, H-3), 3.80 (s, 3H, Me ester), 3.75–3.72 (m, 2H, H-6b + H-4), 3.61 (t, *J* = 9.3 Hz, 1H, H-3′), 3.55 (t, *J* = 7.9 Hz, 1H, H-2′), 3.47 (s, 3H, OMe), 3.30–3.25 (m, 3H, H-5′′ + H-3′′ + H-2′′), 3.20–3.18 (m, 2H, H-2 + H-4′′), 1.92 (s, 3H, *CH*_3_CO). ^13^C-NMR (CDCl_3_) (β anomer) δ: 170.7 (CO, acetyl), 168.2 (CO, Me ester), 129.0–126.2 (Bn), 103.8 (C-1′), 101.6 (C-1′′), 100.6 (C-1), 82.4 (C-3′′), 81.8 (C-3′), 80.8 (C-2′), 79.8 (C-4′′), 78.4 (C-4′), 77.6 (C-3), 76.8 (C-4), 75.5 (CH_2_, Bn), 75.3 (CH_2_, Bn), 74.8 (CH_2_, Bn), 74.4 (C-5′), 73.1 (C-5′′), 72.6 (CH_2_, Bn), 66.4 (C-2′′), 65.0 (C-6), 62.8 (C-6′′), 60.8 (OMe), 59.9 (C-2), 57.0 (*CH*_3_, Me ester), 20.5 (*CH*_3_CO).

*(2-Azido-3-O-benzyl-2-deoxy-4-O-methyl-α-d-glucopyranosyl)-O-(1→4)-(2,3-di-O-benzyl-β-d-gluco-pyranosyluronate)-O-(1→4)-1,6-anhydro-2-azido-3-O-benzyl-2-deoxy-β-d-glucopyranose* (**16**). Lithium hydroxide (0.7 M in H_2_O, 37.3 mL) was added was slowly added to a cooled (−5 °C) solution of **15** (1.35 g, 1.3759 mmol) in THF–MeOH (1:1, 240 mL) containing H_2_O_2_ (41.8 mL). After overnight stirring at RT the mixture was acidified to pH 2 by 1 M HCl. The product was extracted by DCM (200 ml and 80 mL) washed with 5% aqueous Na_2_S_2_O_3_ and brine and dried (Na_2_SO_4_) to give **13** (1.12 g, 82%) after evaporation. ESIMS *m*/*z*: [M − H]^+^: 923.3761 . Calculated for C_47_H_51_N_6_O_14_: 923.3458.

*(2-Azido-3-O-benzyl-2-deoxy-4-O-methyl-6-O-sodiumsulfonato-α-d-glucopyranosyl)-O-(1→4)-(2,3-di-O-benzyl-β-d-sodium glucopyranosyluronate)-O-(1→4)-1,6-anhydro-2-azido-3-O-benzyl-2-deoxy-β-d-gluco-pyrano se* (**17**). SO_3_:Py complex (854 mg, 5.37 mmol) was added to a solution of **16** (0.993 g, 1.07 mmol) in dry pyridine (34 mL) and the reaction mixture was heated under nitrogen atmosphere at 55 °C for 2.0 h. After evaporation the crude product was purified by flash chromatography (DCM–MeOH, 97:3) to give **17** (825 mg, 76%). ^1^H-NMR (500 MHz, CDCl_3_) δ: 7.37–7.24 (m, 20H, Ph), 5.48 (s, 1H, H-1), 5.43 (d, 1H, *J* = 3.85 Hz, H-1′′), 5.03, 4.66 (AB system, 2H, *J* = 10.8 Hz, *CH*_2_Ph), 5.03, 4.80 (AB system, 2H, *J* = 10.8 Hz, *CH*_2_Ph), 4.85 (2H, *CH*_2_Ph), 4.59 (s, 2H, *CH*_2_Ph), 4.57 (s, 1H, H-1′), 4.56 (m, 1H, H-5), 4.20 (dd, 1H, *J* = 2.1 and 11.0 Hz, H-6′′a), 4.17 (m, 2H, H-6′′b + H-6a), 4.00 (t, 1H, *J* = 9.0 Hz, H-4′′), 3.89 (d, 1H, *J* = 7.4 Hz, H-6a), 3.83 (s, 1H, H-5′), 3.80–3.71 (m, 5H, H-3 + H-3′ + H-3′′ + H-4 + H-2′), 3.56 (t, 1H, *J* = 8.45 Hz, H-5′′), 3.52 (s, 3H, OMe), 3.19–3.13 (m, 3H, H-2′′ + H-2 + H-4’). ^13^C-NMR (CDCl_3_): δ: 169.4(C=O), 138.1–137.5 (Ph), 128.1–127.2 (Ph), 103.2 (C-1′), 100.5 (C-1), 97.5 (C-1′′), 83.8 (C-3′), 81.4 (C-2′) 80.0 (C-4′), 79.4 (C-3′), 78.2 (C-3), 76.4 (C-4), 76.0 (C-4′′), 76.1 (CH_2_, Bn), 75.8 (CH_2_, Bn), 74.9 (C-5), 75.2 (C-5′), 73.2 (CH_2_, Bn), 72.5 (C-5′′), 65.1 (C-6), 63.6 (C-2′′), 62.1 (C-6′′), 61.5 (OMe), 60.5 (C-2). ESIMS: [M − H]^+^: 1003.3061. Calculated for C_47_H_51_N_6_O_17_S: 1003.3026.

*(2-Amino-2-deoxy-4-O-methyl-6-O-sodium sulfonato-α-d-glucopyranosyl)-O-(1→4)-(2,3-di-O-benzyl-β-d-sodium glucopyranosyluronate)-O-(1→4)-1,6-anhydro-2-amino-3-O-benzyl-2-deoxy-β-d-glucopyranose* (**18**). Compound **17** (760 mg, 0.7562 mmol) dissolved in t-BuOH–H_2_O (1:1) (76 mL) was stirred under H_2_ in the presence of 10% Pd/C catalyst (760 mg) for 60 h. Filtration through Celite and evaporation of the solvent gave **18** (450 mg, yield 100%). ^1^H-NMR (500 MHz, D_2_O) δ: 5.45 (s, 1H, H-1), 5.44 (s, 1H, H-1′′), 4.78 (d, *J* = 5.5 Hz, 1H, H-5), 4.62 (d, d = 8.0 Hz, 1H, H-1′), 4.29 (dd, *J* = 2.2 and 11.0 Hz, 1H, H-6′′a), 4.16 (dd, *J* = 2.2 and 11.0 Hz, 1H, H-6b), 4.18 (d, *J* = 7.2 Hz, 1H, H-6′′b), 3.94 (t, *J* = 1.5 Hz, 1H, H-3), 3.90 (dt, *J* = 2.1 and 10.1 Hz, 1H, H-5′′), 3.82-3.73 (m, 5H, H-6b + H-3′ + H-4 + H-4′ + H-5′), 3.68 (t, *J* = 9.8 Hz, 1H, H-3′′), 3.58 (s, 3H, OMe ), 3.41 (dd, *J* = 7.8 and 9.2 Hz, 1H, H-2′), 3.28 (t, *J* = 9.7 Hz, 1H, H-4′′), 2.87 (dd, *J* = 4.0 and 10.2 Hz, 1H, H-2′′), 2.82 (s, 1H, H-2). ^13^C-NMR (D_2_O): δ: 179.0 (COOH), 104.9 (C-1), 104.5 (C-1′), 101.2 (C-1′′), 81.4 (C-4′′), 80.7 (C-5′), 79.4 (C-4 + C-4′), 78.8 (C-3′), 77.2 (C-5), 76.0 (C-2′), 75.2 (C-3′′), 73.8 (C-3), 73.1 (C-5′′), 69.1 (C-6′′), 67.8 (C-6), 62.8 (OMe), 57.7 (H-2′′), 55.8 (H-2). ESIMS *m*/*z*: [M − H]^+^: 591.1395. Calculated for C_19_H_31_N_2_O_17_S: 591.1337.

*(2-Deoxy-4-O-methyl-2-sodium sulfonatamido-6-O-sodium sulfonato-α-d-glucopyranosyl-O-(1→4)-(sodium β-d-glucopyranosyluronate)-O-(1→4)-1,6-anhydro-2-deoxy-2-sodium sulfonatamido-β-d-glucopyranose* (**1**). Aqueous sodium hydroxide (2 M) was added till pH 8.5 to a solution of **15** (100 mg, 0.169 mmol) in H_2_O (15 mL). Sulphur trioxide-pyridine complex (970 mg, 6.08 mmol) was then added at RT in five portions in a 1.5 h interval while the pH maintained to 8–9 by addition of 2 M NaOH. After stirring at RT overnight the solution was concentrated to 7 mL and layered on top of a Sephadex G10 column (2.5 cm × 80 cm) eluted by aqueous 0.2 M NaCl. The fractions were collected and concentrated. Compound **1** (81 mg, 57%) was obtained after desalting on Sephadex G 10 and lyophilisation. ^1^H-NMR (500 MHz, D_2_O) δ: 5.63 (s, 1H, H-1), 5.61 (d, *J* = 3.75 Hz, 1H, H-1′′), 4.78 (d, *J* = 5.4 Hz, 1H, H-5), 4.62 (d, *J* = 7.9 Hz, 1H, H-1′), 4.30 (dd, *J* = 2.1 and 11.0 Hz, 1H, H-6′′a), 4.18 (m, 1H, H-6′′b), 4.18 (d, *J* = 11.0, 1H, H-6a), 3.95 (H-3), 3.88 (H-5′′), 3.86 (H-3′), 3.83 (H-5′), 3.82 (H-4), 3.78 (H-4′), 3.80 (m, 1H, H-6b), 3.67 (t, *J* = 9.7 Hz, 1H, H-3′′), 3.58 (s, 3H, OMe), 3.44 (t, *J* = 9.7 Hz, 1H, H-2’), 3.36 (t, *J* = 7.9 Hz, 1H, H-4′′), 3.28 (dd, *J* = 3.8 and 10.5 Hz, 1H, H-2′′), 3.21 (s, 1H, H-2). ^13^C-NMR (D_2_O): 178.6 (COOH), 104.5 (C-1), 103.0 (C-1′), 100.4 (C-1′′), 81.5 (C-4′′), 79.8 (C-3′), 79.6 (C-4′), 79.3 (C-4), 79.1 (C-5′), 76.4 (C-5), 75.4 (C-2′), 73.8 (C-3′′), 73.2 (C-3), 71.5 (C-5′′), 69.2 (C-6′′), 68.1 (C-6), 68.2 (OMe), 60.9 (C-2′′), 58.6 (C-2). LC-MS: [M – H]^+^: 751.0561. Calculated for C_19_H_31_N_2_O_23_S_3_: 751.0474.

*(2-Deoxy-4-O-methyl-2-sodium sulfonatamido-6-O-sodium sulfonato-α-d-glucopyranosyl)-O-(1→4)-(glycol-split sodium β-d-glucopyranosyluronate)-O-(1→4)-1,6-anhydro-2-deoxy-2-sodium_sulfonatamido-β-d-glucopyranose.* Or: *sodium(2S,3S)-4-hydroxy-2-((R)-2-hydroxy-1-(((1R,2S,3R,4R,5R)-3-hydroxy-4-(sulfonatoamino)-6,8-dioxabicyclo[3.2.1]octan-2-yl)oxy)ethoxy)-3-(((2R,3R,4R,5S,6R)-4-hydroxy-5-methoxy-3-(sulfonatoamino)-6-((sulfonatooxy)methyl)tetrahydro-2H-pyran-2-yl)oxy)butanoate* (**2**). An aqueous solution of sodium metaperiodate (0.2 M, 2.1 mL) was added at RT to a solution of **1** (55 mg, 0.0731 mmol) in H_2_O (2.1 mL). After 5 h, ethylene glycol (0.24 mL, 4.4 mmol) was added and the solution was stirred at RT for 1 h (N.B.: light must be avoided during periodate oxidation). Sodium borohydride (100 mg, 2.633 mmol) was then added in portions at 0 °C. After 16 h, HOAc (0.175 mL) was added at 0 °C to pH 7. After concentration the salt were removed using a Sephadex G10 column eluted by 10% EtOH–H_2_O. Lyophilisation gave **2** (35.2 mg, 70.4%). ^1^H-NMR (500 MHz, D_2_O) δ: 5.59 (s, 1H, H-1), 5.34 (d, *J* = 3.65 Hz, 1H, H-1′′), 4.86 (t, *J* = 4.59 Hz, 1H, H-1′), 4.69 (d, *J* = 5.45 Hz, 1H, H-5), 4.29 (dd, *J* = 6.0 and 11.0 Hz, 1H, H-6′′a), 4.21 (m, 1H, H-6′′b), 4.16 (d, *J* = 6.0 Hz, 1H, H-4), 4.15 (m, 1H, H-6a), 3.97 (d, *J* = 8.0 Hz, 1H, H-5′′), 3.94 (m, 1H, H-4′), 3.94 (m, 1H, H-3), 3.92 (m, 1H, H-3′a), 3.90 (m, 1H, H-5′), 3.73 (m, 1H, H-6b), 3.80 (m, 1H, H-3′b), 3.68 (m, 1H, 1H, H-3′′), 3.66 (m, 2H, H-2′a, H2′b), 3.58 (s, 3H, OMe), 3.35 (t, *J* = 9.75 Hz, 1H, H-4′′), 3.29 (dd, *J* = 3.65 and 10.45 Hz, 1H, H-2′′), 3.20 (s, 1H, H-2). ^13^C-NMR (D_2_O) δ: 179.58 (COOH), 104.2 (C-1), 103.9 (C-1′), 98.4 (C-1′′), 81.8 (C-4′′), 80.8 (C-4′), 80.5 (C-4), 77.8 (C-5′), 76. 5 (C-5), 73.8 (C-3′′), 73.4 (C-3), 71.8 (C-5′′), 69.2 (C-6′′), 67.9 (C-6), 64.6 (C-2′), 62.6, (OCH_3_), 61.8 (C-3′), 60.8 (C-2′′), 58.3 (C-2). LC-MS: [M − H]^+^: 753.0884. Calculated for C_19_H_31_N_2_O_23_S_3_: 753.0708.

### 3.3. MM/MD Simulation

Models for **1** and **2** are built by Maestro/Macromodel 9.8 software where GlcNS6S and GlcA are drawn in ^4^C_1_ conformation while the 1,6anGlcNS is built in ^1^C_4_ conformation, as previously determined [24,25]. The conformation of the glycol-split GlcA residue is initially guessed by visual inspection. Two different initial conformations for both **1** and **2** are built, characterized by two sets of dihedral angles values. In **2** in going from non reducing to the reducing end, the dihedrals are defined as follows: τ (C-O4-C4-H4 between MeO and GlcNS6S), α/β (H1-C1-O4-C4/C1-O4-C4-H4 between GlcNS6S and gsGlcA), γ (H4-C4-C5-H5 in gsGlcA), δ/ε (H5-C5-O5-C1/C5-O5-C1-H1 in gsGlcA), ω/w (H1-C1-O4-C4/C1-O4-C4-H4 between gsGlcA and 1,6anGlcNS). In **1** only five dihedral angles are defined: τ, α/β, and ω/w. The Amber* force field as implemented in Maestro/Macromodel 9.8 software is used, the non-bonded cut-off are set to 20.0, 8.0 and 4.0 Å for electrostatic, Van der Waals and hydrogen bond interactions respectively. The solvent description involve the Generalized Born Implicit solvent method. The glycan models after building are energy minimized (bmin procedure) setting: Max number of steps = 10 K, and Gradient Threshold = 10^−3^ KJ·mol^−1^·Å^−1^. Each of the four glycan models is then submitted to a sequence of eleven MD simulation run with temperature progressively increasing from 300 to the highest temperature value of 400 K, using steps of 20 K, to decrease again to the final value of 300 K. The time length of each fixed temperature MD run is 5 ns for a whole duration of 55 ns. The MD simulation thermal history is summarized in Table S1. The glycan backbone torsional angles at the beginning and at the end of the MD sampling method, averaging for a suitable amount of time in the final MD run, are reported in Table S2. To simulate **2** and **1** 2D NOEs signals the NOEPROM (Martin-Pastor, 2005) software is used, while for both glycans the correlation time (Tc = 350 ps, isotropic model of motion) is estimated reproducing qualitatively the H-1/H-2 and H-1′′/H-2′′ NOEs build up curves in the mixing time range between 0.2 to 1.0 or 1.5 s, for GlcNS6S and 1,6anGlcNS residues as reported in Supplementary Materials Figure S5. Selected interglycosidic NOEs for **1** and **2** measured in the mixing time range between 0.2 to 1.0 or 1.5 s are reported in Supplementary Materials Figure S6, with the corresponding properties simulated using the glycan predicted conformation. The point-like charge distribution are estimated in accord to the AM1-BCC approach, using the antechamber routine included in Amber tools 1.4 software (Case, D.A. et al., 2010, AMBER 11, University of California San Francisco, San Francisco, CA, USA).

## 4. Conclusions

To investigate the role of glycol-split uronic acid units in glycol-split-heparin-derived inhibitors of heparanase we have synthesized trisaccharide **1** and its glycol-split counterpart **2**. In an heparanase inhibition assay **2** was found one order magnitude more active than the ring closed **1**. The activity of **1** and **2** is however low (IC_50_ in the µM range) in comparison to roneparstat. In parallel a conformational characterization of compound **1** and **2** is reported using NOESY NMR and MD simulations. The GlcA ring opening introduces three additional dihedral angles: γ, δ, ε, improving the backbone degree of freedom. The most populated backbone dihedral angle states for both compound **1** and **2** are estimated, using gradient temperature MD simulation, and selected inter-glycosidic NOEs enhancements as constraint. The glycol-split trisaccharide is characterised by a greater flexibility, a smaller end-to-end distance, and a different electric charge distribution.

## Supplementary Materials

Supplementary materials can be accessed at: http://www.mdpi.com/1420-3049/21/11/1602/s1.
