# Supplementary material for: Investigating Glycol-Split-Heparin-Derived Inhibitors of Heparanase: A Study of Synthetic Trisaccharides
Source: Molecules. 2016 Nov 23;21(11):1602. doi: 10.3390/molecules21111602 (PMC6274180; doi:10.3390/molecules21111602)
Supplement: Supplementary file 1 [file molecules-21-01602-s001.pdf]

Supplementary Materials: Investigating Glycol-Split-Heparin-Derived Inhibitors of Heparanase: Study of Synthetic Trisaccharides

Minghong Ni, Stefano Elli, Annamaria Naggi, Marco Guerrini, Giangiacomo Torri and Maurice Petitou

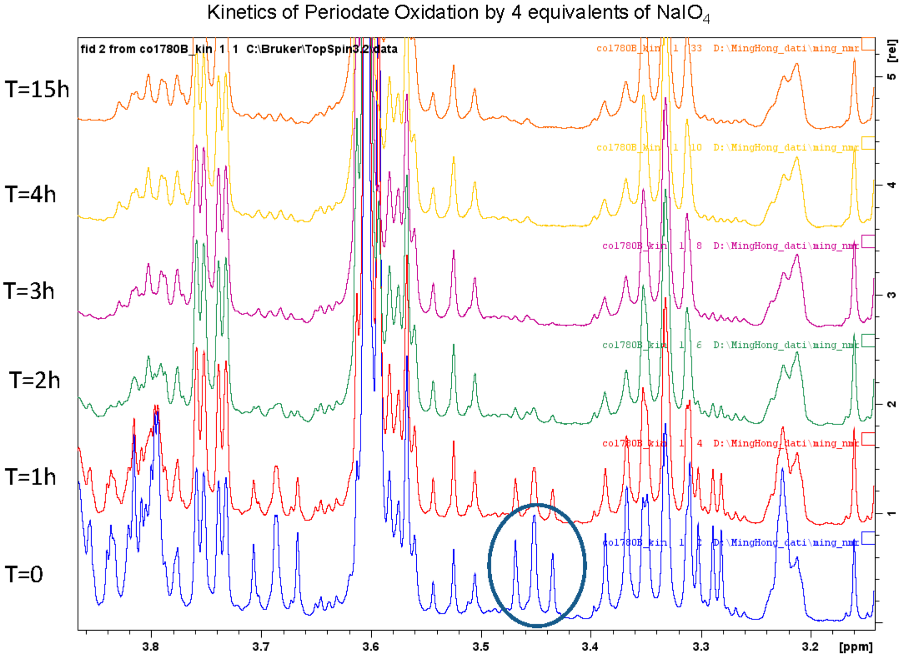

Figure S1. ^{1}H-NMR spectra used to monitor the periodate oxidation of **1**. The circled signal that disappears throughout the reaction corresponds to H-2′ of GlcA (3.44 ppm).

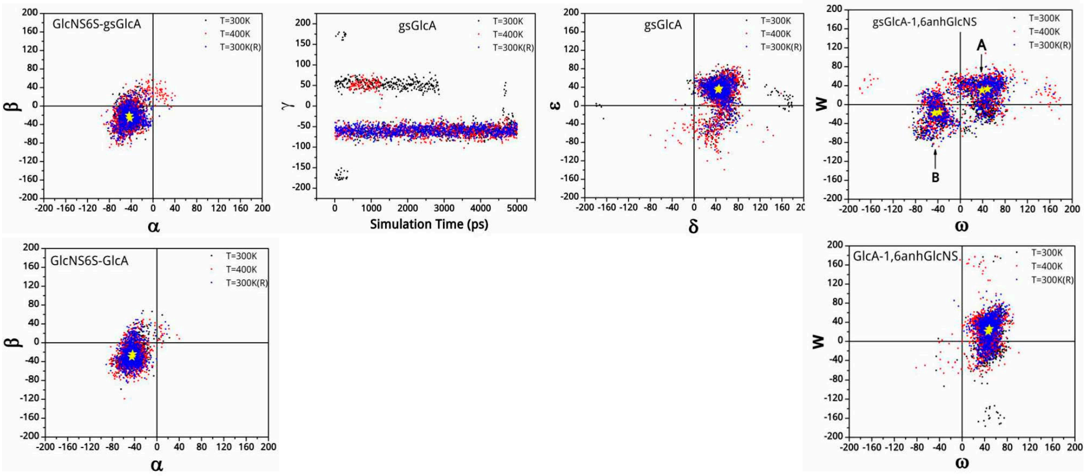

Figure S2. Backbone computed dihedral angles: α/β, γ, δ/ε, ω/w for compound **2** (top) and the comparable α/β, ω/w for compound **1** (bottom) sampled during selected MD simulations steps at temperature 300 K (step 1, black dots), 400 K (step 6, red dots) and 300 K (step 11, blue dots). The dihedral angles pairs: α/β, γ, δ/ε, ω/w are reported in Ramachandran diagrams, while γ is reported as a function of simulation time. Two possible conformations for compound **2**, characterized by different glycosidic state ω/w (see Table S2) are found (A, and B). Yellow stars in Ramachandran plots indicate torsional states defined by averaging the corresponding dihedral angle pairs for a suitable amount of simulation time.

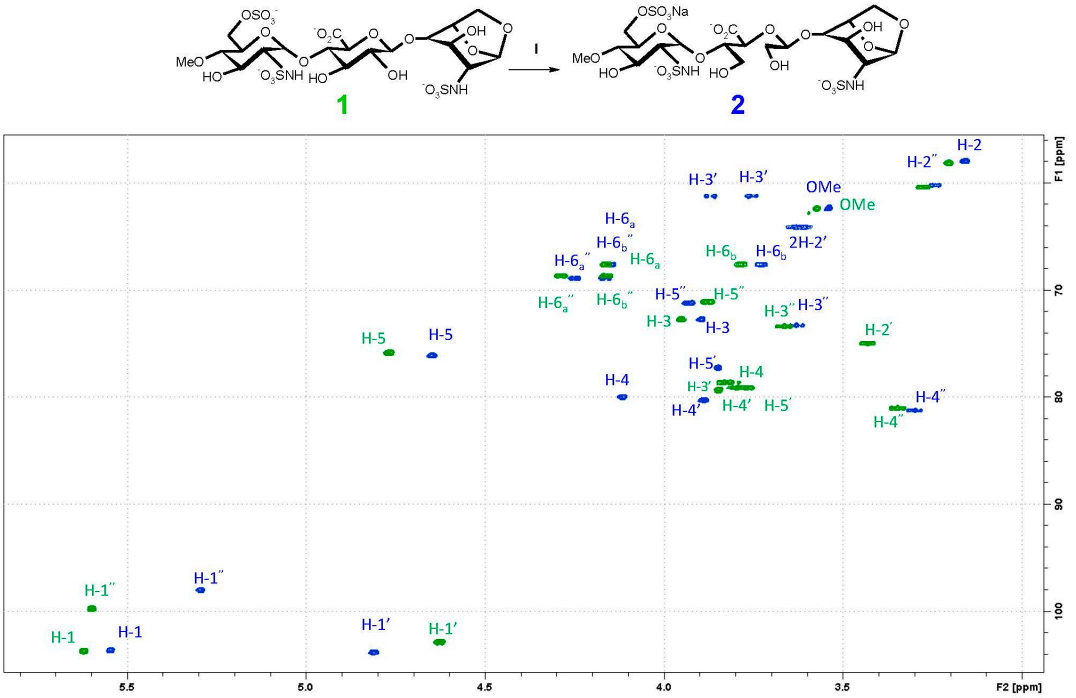

Figure S3. 2D HSQC spectra of compound **1** (green) and **2** (blue). (no quote, quote and double quote respectively refer to reducing-end unit, central unit and non reducing end unit).

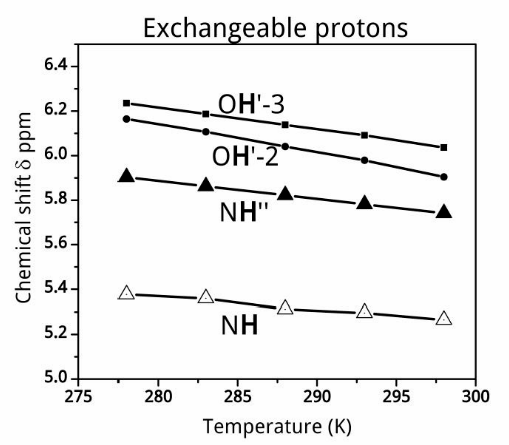

Figure S4. Plot of the chemical shift of exchangeable protons in **2** vs. temperature (K).

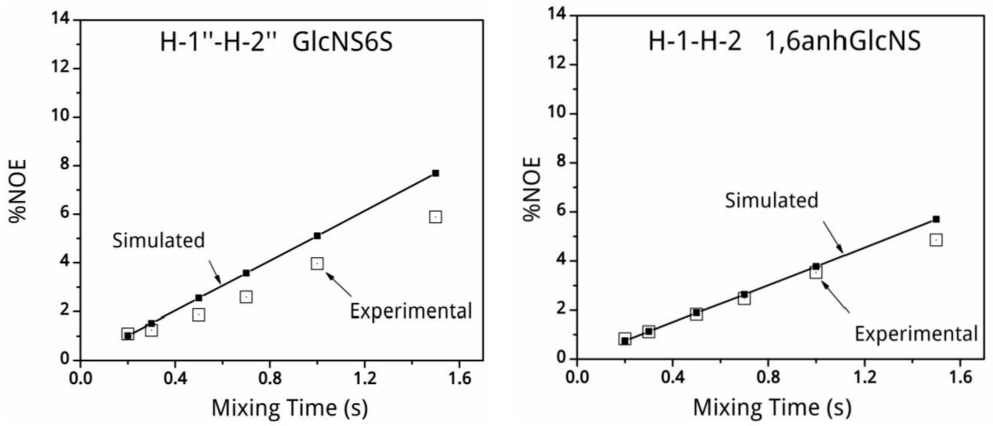

Figure S5. Intra-residue experimental (empty symbols) and simulated (black line) 2D NOEs build up curves for the glucosamine units in **2**. The simulation was performed using the A conformer (see text).

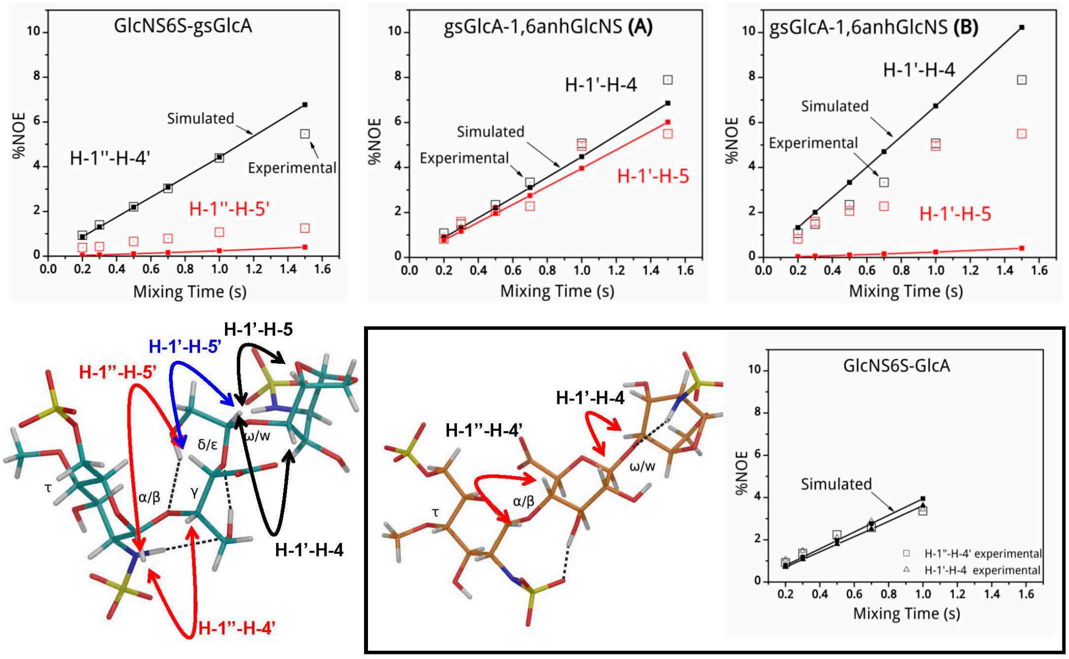

Figure S6. Selected 2D NOEs. Upper part: Selected inter-glycosidic 2D NOEs build up curves for the two (see Figure S2, **A** and **B**) predicted conformations of **2** at mixing time between 0.2 to 1.5 s. Comparison of the middle and right panels shows the better fit obtained between experimental and modelled curves when the **A** conformer is used for the calculation. Lower part: A licorice representation of **2** in conformation **A** showing the previously discussed inter-residue NOEs. On the right a good fit is obtained between experimental and calculated NOEs for the obtained conformation of **1**.

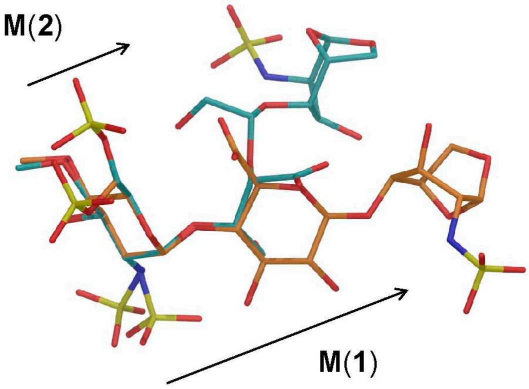

Compound **2** has a shorter dipole moment vector than **1**.

Dipole moment vector

Molecule	Mx	My	Mz	|**M**| (e Å)
**2** (A)	2.8	-2.1	-1.2	**3.7**
**2** (B)	-0.9	2.6	-2.9	**4.0**
**1**	4.3	3.9	-4.7	**7.4**

Figure S7. Distribution charge properties: dipole moment lenght and components, estimated for **1** and **2** in the previously determined conformations using the approach AM1-BCC (see text). On the left, the non-reducing end residues of **1** and **2** have been superposed.

Table S1. Summary of the MD simulation thermal history for the model of compound **1** and **2**.

MD Simulation Step	1	2	3	4	5	6	7	8	9	10	11
Temperature (K)	300	320	340	360	380	400	380	360	340	320	300
Time Length (ns)	5K	5K	5K	5K	5K	5K	5K	5K	5K	5K	5K

Table S2. Backbone torsional angles for **1** and **2**. Values in **black** refer to the two initial conformations setted for each glycans (**1.1–1.2** and **2.1–2.2)** (black coloured lines). Values in red are the torsional angles obtained. Compound **2** significantly populates two states (A and B) at the torsional degree of freedom ω/w.

		**Torsional Angles (°)**				
		**τ**	**α/β**	**γ**	**δ/ε**	**ω/w**
1	Initial 1.1	−20	−40/−26	–	–	53/9
	**Final 1.1**	**−17**	**−44/−24**	**–**	**–**	**49/26**
	Initial 1.2	−60	−20/26	–	–	−30/−30
	**Final 1.2**	**−15**	**−45/−30**	**–**	**–**	**46/21**
2	Initial 2.1	−61	−52/−26	180	50/51	56/7
	**Final 2.1**	**−19**	**−42/−27**	**−59**	**44/34**	**49/33 (A) −37/−16 (B)**
	Initial 2.2	180	−30/−50	61	0/30	−40/10
	**Final 2.2**	**−20**	**−44/−20**	**−69**	**46/37**	**39/30 (A) −46/−18 (B)**

Table S3. Temperature coefficients Δδ (ppb·K^{-1}) estimated by linear regression. The estimated errors (ΔΔδ) is shown on the last decimal digit. The linear correlation coefficient (*R*) is reported

**H-Bond**	**Δδ (ΔΔδ)**	***R***
OH′-3	−9.8 (2)	−0.9996
OH′-2	−13.0 (3)	−0.9991
NH″	−8.09 (3)	−0.99997
NH	−5.7 (5)	−0.9891
